# Integrative Omics Analysis Reveals Mechanisms of Anthocyanin Biosynthesis in Djulis Spikes

**DOI:** 10.3390/plants14020197

**Published:** 2025-01-12

**Authors:** Chunmei Zheng, Wenxuan Ge, Xueying Li, Xiuzhang Wang, Yanxia Sun, Xiaoyong Wu

**Affiliations:** Key Laboratory of Coarse Cereal Processing, Ministry of Agriculture and Rural Affairs, Sichuan Engineering and Technology Research Center of Coarse Cereal Industrialization, School of Food and Biological Engineering, Chengdu University, Chengdu 610106, China; zhengchunmei@stu.cdu.edu.cn (C.Z.); gewenxuan@stu.cdu.edu.cn (W.G.); lxy374529650@163.com (X.L.); wxz13864756970@163.com (X.W.)

**Keywords:** *Chenopodium formosanum* Koidz, anthocyanins targeted metabolomics, transcriptomics, untargeted metabolomics

## Abstract

Djulis (*Chenopodium formosanum* Koidz.), a member of the *Amaranthaceae* family plant, is noted for its vibrant appearance and significant ornamental value. However, the mechanisms underlying color variation in its spikes remain unexplored. This research initially detected the anthocyanin content at different developmental stages of the spike and subsequently utilized an integrative approach, combining targeted metabolomics, transcriptomics, and untargeted metabolomics analyses, to elucidate the mechanisms of anthocyanin biosynthesis in the spikes of djulis. The results of the combined multi-omics analysis showed that the metabolites associated with anthocyanin synthesis were mainly enriched in the flavonoid biosynthesis pathway (ko00941) and the anthocyanin biosynthesis pathway (ko00942). With the maturation of djulis spikes, a total of 28 differentially expressed genes and 17 differentially expressed metabolites were screened during the transition of spike color from green (G) to red (R) or orange (O). Twenty differentially expressed genes were selected for qRT-PCR validation, and the results are consistent with transcriptome sequencing. The upregulation of seven genes, including *chalcone synthase* (*CfCHS3_1*, *CfCHS3_2*, *CfCHS3_3*), *flavanone 3-hydroxylase* (*CfF3H_3*), *flavonoid 3′5′-hydroxylase* (*CfCYP75A6_1*), *dihydroflavonol reductase* (*CfDFRA*), and *glucosyltransferase* (*Cf3GGT*), promotes the formation and accumulation of delphinidin 3-sambubioside and peonidin 3-galactoside. The research results also showed that anthocyanins and betalains can coexist in the spike of djulis, and the reason for the change in spike color during development may be the result of the combined action of the two pigments. A possible regulatory pathway for anthocyanin biosynthesis during the spike maturation was constructed based on the analysis results. The results provide a reference and theoretical basis for further studying the molecular mechanism of anthocyanin regulation of color changes in *Amaranthaceae* plants.

## 1. Introduction

Djulis (*Chenopodium formosanum* Koidz.) is a pseudo-cereal crop growing in Taiwan, China, and is predominantly situated in Pingtung and Taitung counties, with an elevation spanning from 230 to 1568 m [1]. It is an annual dicotyledonous herb belonging to the genus *Quinoa* in the family *Amaranthaceae* [2,3]. Djulis, with shallow roots and spikes, can grow vertically to a height of two meters or more, with its primary and side branches yielding seeds [4]. Djulis demonstrates robust stress resistance, exhibiting remarkable traits such as tolerance to cold, drought, and salinity [4,5]. As a grain crop, djulis is notable for its high nutritional value and numerous beneficial biological effects. Additionally, its vibrant red spike color enhances its ornamental appeal, earning it the moniker “ruby of grains” [6]. During the developmental phase, the djulis spikes exhibit a green coloration, which transitions to a red or orange hue upon maturation, thereby enhancing their visual appeal.

The seeds of djulis are abundant in a range of nutrients, including dietary fiber, starch, polyphenolic compounds such as gallic acid, flavonoids such as rutin, proteins, essential amino acids, unsaturated fatty acids, minerals, and vitamins [7,8,9]. Owing to this rich functional composition, djulis and its bioactive compounds exhibit various physiological functions, such as hypoglycemic, diabetes prevention [10,11,12], hypertension prevention [13], hypolipidemia [14,15], colon cancer prevention [16], liver protection [17,18], inducing apoptosis of hepatocellular carcinoma cells [19,20], repairing skin damage and anti-aging [7,21,22], and so on. In addition, djulis has traditionally been used as an insect repellent [23]. A study by Chio et al. [24] evaluated the toxicity and repellence of djulis against mosquitoes and biting midges using methanol or dichloromethane, both containing 9, 12-octadecadienoyl chloride (Z, Z) in the compounds extracted from djulis. The study by Chuang et al. [25], on the other hand, demonstrated that the effective insecticidal effect of the ethanol extract of djulis was attributed to 1,2-benzenediol, 5-HMF (5-hydroxymethylfurfural), and 2-heptanone. Owing to its remarkable resistance, beneficial biological properties, and substantial nutritional content, djulis holds significant potential for applications and market value. Consequently, it has emerged as a prominent subject of research within the scientific community.

**Figure 1:**
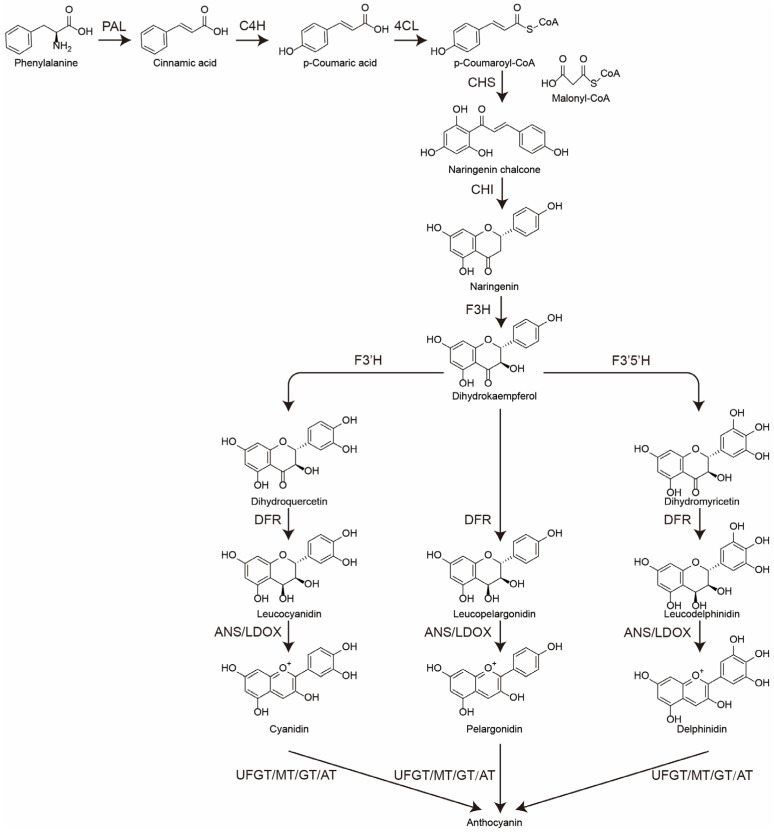
Anthocyanin biosynthesis pathway. PAL: phenylalanine ammonia-lyase; C4H: cinnamate-4-hydroxylase; 4CL: 4-coumarate-CoA ligase; CHS: chalcone synthase; CHI: chalcone isomerase; F3H: flavanone 3-hydroxylase; F3′H: flavonoid 3′-hydroxylase; F3′5′H: flavonoid 3′,5′-hydroxylase; DFR: dihydroflavonol 4-reductase; ANS: anthocyanidin synthase; LDOX: leucoanthocyanidin dioxygenase; UFGT: flavonoid-glucosyl transferase; MT: methyltransferase; GT: glucosyltransferase; AT: acyltransferase.

Anthocyanins are natural water-soluble pigments prevalent in the leaves, fruits, and flowers of plants and are responsible for these vivid colors. Currently, the biosynthetic pathway of anthocyanins has been extensively studied (Figure 1). Anthocyanin biosynthesis originates from the phenylpropanoid pathway, specifically during the phenylalanine metabolism stage. This process primarily involves the production of anthocyanins through the activities of phenylalanine ammonia-lyase (PAL), cinnamate-4-hydroxylase (C4H), and 4-coumarate-CoA ligase (4CL), which ultimately lead to the formation of coumaroyl CoA [26]. Subsequently, chalcone synthase (CHS), chalcone isomerase (CHI), and flavanone 3-hydroxylase (F3H) catalyze reactions that result in the production of dihydrokaempferol, a crucial dihydroflavonol compound [27,28]. Flavonoid 3′-hydroxylase (F3′H) and flavonoid 3′,5′-hydroxylase (F3′5′H), both members of the cytochrome P450 superfamily, catalyze the production of dihydroquercetin and dihydromyricetin, respectively, utilizing dihydromyricetin as a substrate [29,30]. Subsequently, dihydroflavonol 4-reductase (DFR) facilitates the conversion of dihydrocannabinol, dihydroquercetin, and dihydromyricetin into the colorless compounds pelargonidin, cyanidin, and delphinidin, respectively [31]. Anthocyanidin synthase (ANS), also known as leucoanthocyanidin dioxygenase (LDOX), represents a crucial gene involved in the latter stages of anthocyanin biosynthesis, facilitating the conversion of colorless anthocyanidins into anthocyanins [32]. Subsequently, the carbon skeleton of anthocyanins undergoes modifications through methylation, glycosylation, and acylation, resulting in the formation of stable anthocyanins. These modifications are mediated by enzymes such as flavonoid-glucosyl transferase (UDP-glucose flavonoid 3-O-glucosyl transferase, UFGT), methyltransferase (MT), glucosyltransferase (GT), and acyltransferase (AT), among others [33,34]. In nature, anthocyanins are primarily categorized into six major classes: delphinidins, cyanidins, pelargonidins, peonidins, petunidins, and malvidins [35]. Among them, petunidins and malvidins can be derived from the methylation of delphinidin, and peonidins are generated from the methylation of cyanidins [34].

Numerous studies have been conducted on anthocyanins and the key structural genes across various species. Shi et al. [36], in their investigation of color formation in the fruit skin of Chinese jujube (*Ziziphus jujuba* Mill.), identified that cyanidin-3-O-rutinoside and peonidin-3,5-O-diglucoside are the predominant anthocyanins, with *ZjANS* and *ZjUGT79B1* being crucial genes involved in anthocyanin biosynthesis. Similarly, Zhong et al. [37] examined the coloration of the bark of the camphor tree (*Cinnamomum camphora*) and determined that cyanidin, pelargonidin, and peonidin were the primary anthocyanin components. The transcript abundance of *PAL*, *4CL*, *CHS*, *F3H*, *F3′H*, *UGT*, and *OMT* was significantly higher in the red bark compared to the green bark. Zhang et al. [38] concluded that pelargonidin 3-O-glucoside and pelargonidin 3-O-(6-O-malonyl-β)-D-glucoside were the primary compounds responsible for the reddish coloration of the petal margins in Carnation (*Dianthus caryophyllus*), with *DFR* and *ANS* exhibiting significantly increased expression during the discoloration process. Wang et al. [39] identified that the dark red blotches on the yellow petals of Rosa persica result from the accumulation of cyanidin, pelargonidin, and peonidin, with *4CL*, *DFR*, *ANS*, and *UFGT* being the key structural genes for anthocyanin synthesis. Similarly, Chen et al. [40] reported the accumulation of petunidin-3-O-diglucoside-5-O-glucoside in Sichuan *Fritillaria* (*F. unibracteata*). leaves significantly increased the expression of *F3′5′H* (*Unigene_030074 and Unigene_034772*). Anthocyanins, a significant class of flavonoid secondary metabolites synthesized by plants, exhibit a wide range of biological effects [41,42,43]. Extensive research has demonstrated that anthocyanins possess free radical scavenging and antioxidant properties [44], as well as anti-inflammatory [45] and antibacterial activities [46]. Furthermore, anthocyanins have been shown to alleviate visual fatigue [47], provide renal protection [48], and protect the cardiovascular and cerebrovascular systems [49]. Additionally, they exhibit anticancer properties [50]. These attributes underscore their substantial applicability and have consistently been a focal point of scientific research.

At present, the mechanisms underlying anthocyanin biosynthesis in djulis remain inadequately understood. Previous studies have shown that anthocyanins and betalains in amaranth plants cannot coexist [51]. Genomic data for djulis was made available in 2022 [52]. Consequently, we employed an integrative approach combining metabolomics and transcriptomics analyses to investigate the potential pathways involved in anthocyanin formation in djulis spikes. Our findings indicate that two anthocyanins, delphinidin 3-sambubioside and peonidin 3-galactoside, accumulate in the spikes of djulis during the color transition from green to red or orange. Seven structural genes, *CfCHS3_1*, *CfCHS3_2*, *CfCHS3_3*, *CfFLS_3*, *CfCYP75A6_1*, *CfDFRA*, and *Cf3GGT*, have been identified as potential key regulators in the biosynthesis of two specific anthocyanins. The results of this study contribute to a deeper understanding of the molecular mechanisms underlying anthocyanin accumulation during the discoloration process of djulis spikes. Finally, our study can also serve as a typical case for further exploring whether anthocyanins and betalains coexist.

## 2. Results

### 2.1. Changes in Anthocyanin Content During Spike Development

**Figure 2:**
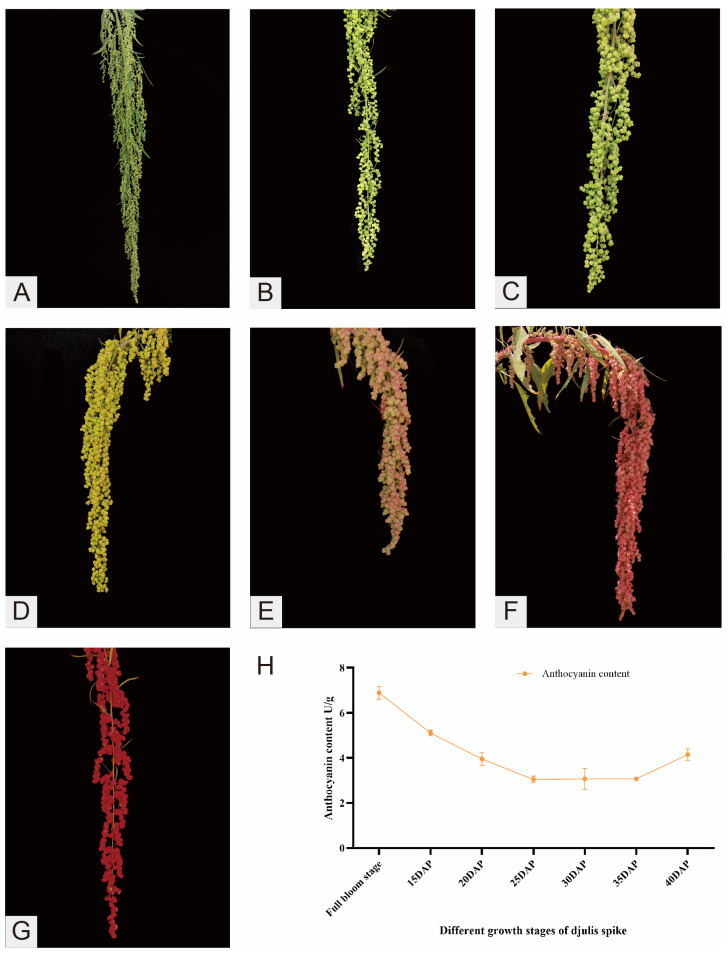
Detection of anthocyanin content. (**A**–**G**) Seven stages of spike development in djulis. The labels (**A**–**G**) represent seven developmental phases, specifically from the full bloom stage to 15, 20, 25, 30, 35, and 40 days after pollination (DAP). (**H**) Line graph of changes in anthocyanin content in djulis spikes at different developmental stages.

The seven stages of spike development in djulis are illustrated in Figure 2A–G. The findings, as depicted in Figure 2H, indicated that anthocyanin content varies throughout spike development. The highest concentration of anthocyanins was observed at the full bloom stage. As the filling stage commenced, there was a gradual decline in anthocyanin content. Subsequently, as the spikes mature and turn red, the anthocyanin content gradually increases again.

**Figure 3:**
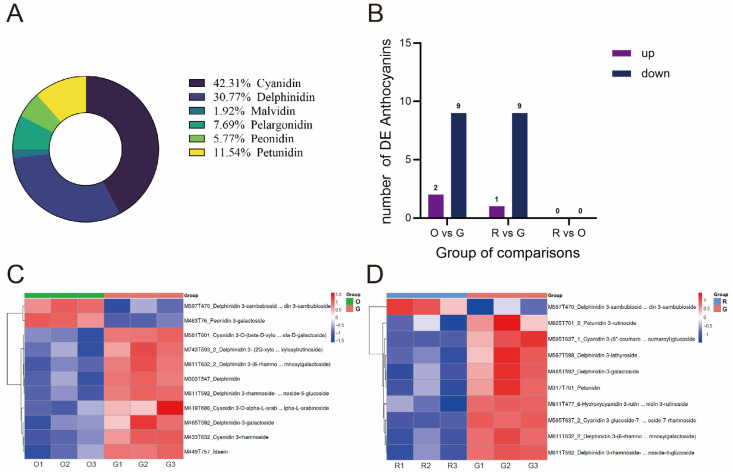
Targeted metabolomics analysis. (**A**) Pie chart of anthocyanin full identification classification.by targeted metabolome. (**B**) Different anthocyanin quantities in different comparison combinations (**C**) Heatmap of O vs. G differential anthocyanin hierarchical clustering. (**D**) Heatmap of R vs. G differential anthocyanin hierarchical clustering.

To further determine the presence of anthocyanins in djulis, the experiment selected green (G), red (R), and orange (O) djulis spike tissues as samples, and performed three biological replicates at each color stage. The targeted metabolome results indicate that 51 distinct species and 6 primary classes of anthocyanins were identified. As illustrated in Figure 3A, the study identified several anthocyanin pigments, including cyanidin, delphinidin, malvidin, pelargonidin, petunidin, and peonidin. These pigments constituted 42.31%, 30.77%, 1.92%, 1.92%, 1.92%, and 1.54% of the total metabolites, respectively. The thresholds applied for screening differential anthocyanins were a *p*-value of <0.05 and a Variable Importance in Projection (VIP) score of >1. In the comparative analyses of O vs. G and R vs. G, 11 and 10 differential anthocyanins were identified, respectively (Figure 3B). There is no difference in anthocyanin in the R vs. O. Among these, two anthocyanins were up-regulated in the O vs. G comparison, while one was up-regulated in the R vs. G comparison. In both comparisons, nine anthocyanins were down-regulated, as illustrated in Figure 3C,D. Specifically, delphinidin 3-sambubioside and peonidin 3-galactoside exhibited up-regulated expression in the O vs. G comparison, whereas delphinidin 3-sambubioside was the sole anthocyanin up-regulated in the R vs. G comparison.

Through anthocyanin content detection and targeted anthocyanin metabolomics analysis, we can find that there are changes in anthocyanins during the transition from green to red or orange in the spikes of djulis. Specifically, during the flowering period and the subsequent green period to the red or orange period after maturity, the content of most anthocyanins decreases, while only a small amount of anthocyanin content increases with color changes.

### 2.2. Non-Targeted Metabolomics

**Figure 4:**
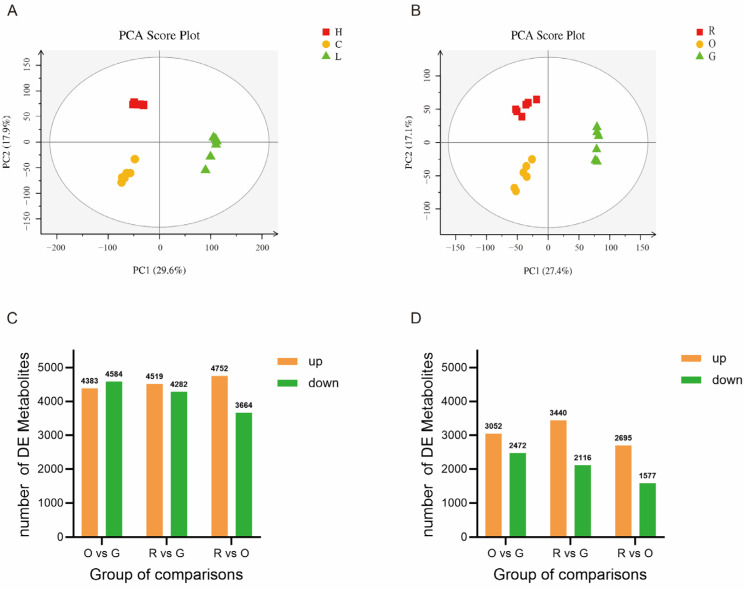
Non-targeted metabolomics analysis. (**A**) PCA score plot in positive ion mode. (**B**) PCA score plot in negative ion mode. (**C**) The number of differential metabolites in each comparison combination under positive ion mode. (**D**) The number of differential metabolites in each comparison combination under negative ion mode.

In the detection of non-targeted metabolomics, six biological replicates were selected from green (G), red (R), and orange (O) djulis spike tissues as samples. Following batch normalization of the peak area data in the metabolomics analysis, a total of 20,036 precursor molecules were identified in the positive ion mode, while 12,430 precursor molecules were identified in the negative ion mode. Perform principal component analysis (PCA) on the data, and observe the clustering of samples within the group and the dispersion of samples between groups from the PCA score graph, indicating good data repeatability and reliability (Figure 4A,B). Perform orthogonal projections to latent structures discriminant analysis (OPLS-DA) on the data, using *p*-value ≤ 0.05 and VIP ≥ 1 as screening thresholds to screen for differential metabolites. In positive ion mode, a total of 8967, 8801, and 8416 metabolites were identified in the comparison groups O vs. G, R vs. G, and R vs. O, respectively (Figure 4C). Among these, 4383, 4519, and 4752 metabolites were up-regulated, while 4584, 4282, and 3664 metabolites were down-regulated. In negative ion mode, 5524, 5556, and 4727 metabolites were detected in the comparisons of O vs. G, R vs. G, and R vs. O, respectively (Figure 4D). Among these, 3052, 3440, and 2695 metabolites were up-regulated, while 2472, 2116, and 1577 metabolites were down-regulated, as illustrated.

The fragmentation data acquired from the MS/MS model were subsequently cross-referenced with annotations in databases such as Metlin (http://metlin.scripps.edu (accessed on 9 January 2025)) and MoNA (https://mona.fiehnlab.ucdavis.edu (accessed on 9 January 2025)) to achieve precise metabolite identification. Following the identification of all measured molecular weights, the detected metabolites were then functionally categorized according to the classification of metabolites in KEGG and Metabolon, Inc. The enrichment analysis of 518 differential metabolites across metabolic pathways revealed that 62, 63, and 57 pathways were significantly enriched in the O vs. G, R vs. G, and R vs. O comparison groups, respectively. Notably, a diverse array of anthocyanin species was enriched within the anthocyanin biosynthesis pathway, which is associated with color variation. In the comparison between O and G, the expression levels of two anthocyanins, peonidin-3-glucoside and delphinidin-3-(p-coumaroyl)-rutinoside-5-glucoside, were found to be up-regulated. Conversely, the expression of three anthocyanins—cyanidin 3-O-beta-D-glucoside 5-O-(6-coumaroyl-beta-D-glucoside), cyanidin 3-O-rutinoside 5-O-beta-D-glucoside, and cyanidin-3-(p-coumaroyl)-rutinoside-5-glucoside—was down-regulated. In the comparison between R and G, the expression levels of five anthocyanins—namely, delphinidin 3-O-beta-D-glucoside 5-O-(6-coumaroyl-beta-D-glucoside), delphinidin 3,7-di-O-beta-D-glucoside, peonidin-3-glucoside, cyanidin 5-O-beta-D-glucoside 3-O-beta-D-sambubioside, and delphinidin-3-(p-coumaroyl)-rutinoside-5- glucoside—were upregulated. Conversely, the expression levels of two anthocyanins, cyanidin 3-O-beta-D-glucoside 5-O-(6-coumaroyl-beta-D-glucoside) and cyanidin 3-O-rutinoside 5-O-beta-D-glucoside, were downregulated. In the comparison of R versus O, there were three anthocyanins—delphinidin 3-O-beta-D-glucoside 5-O-(6-coumaroyl-beta-D-glucoside), delphinidin 3,7-di-O-beta-D-glucoside, and cyanidin-3-(p-coumaroyl)-rutinoside-5-glucoside—that were up-regulated, whereas two anthocyanins, peonidin-3-glucoside and cyanidin 3-rutinoside, were down-regulated. Additionally, the peonidin-3-glucoside and delphinidin-3-(p-coumaroyl)-rutinoside-5-glucoside were up-regulated in both comparative analyses of O versus G and R versus G. The results indicate a variation in anthocyanin-like metabolites associated with the color transition of djulis spikes from green to red or orange.

### 2.3. Transcriptomics, GO, and KEGG Enrichment Analysis

**Figure 5:**
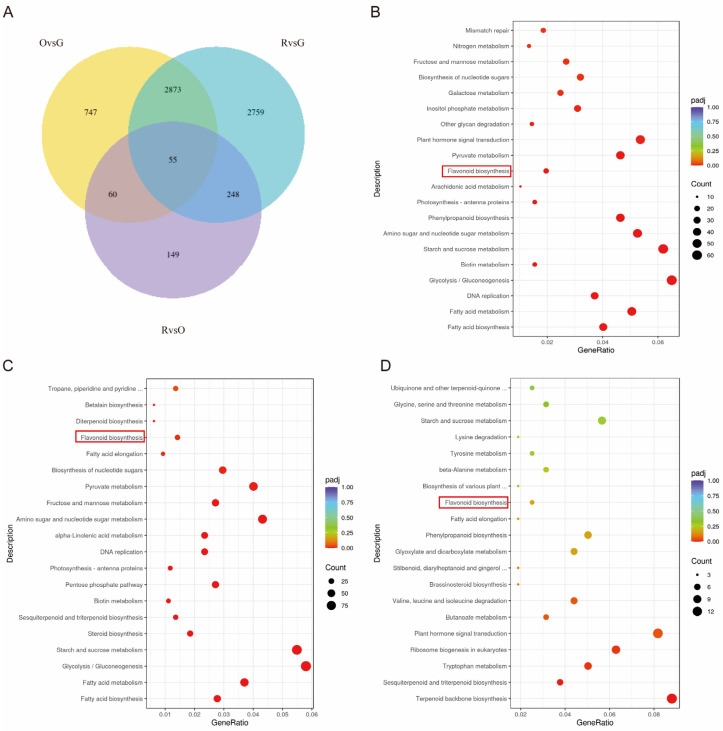
Transcriptomics analysis. The red box in the figure represents the flavonoid biosynthesis. (**A**) Differential gene Wayne plots. (**B**) O vs. G KEGG enrichment scatter plot. (**C**) R vs. G KEGG enrichment scatter plot. (**D**) R vs. O KEGG enrichment scatter plot.

In transcriptome analysis, three biological replicates were selected from green (G), red (R), and orange (O) djulis spike tissues as samples. After eliminating junctions and low-quality bases, a total of 59.61 GB of data was acquired post-quality control. The proportion of Q20 bases exceeded 97.03%, while Q30 bases constituted more than 91.95% of the total bases. Additionally, the measured GC content was greater than 44.04%. The criteria for identifying differentially expressed genes were set at |log2(FC)| ≥ 1 and a padj ≤ 0.05. In the comparative analysis of the three group combinations—O vs. G, R vs. G, and R vs. O—the numbers of differentially expressed genes were 3735, 5935, and 512, respectively. Among these, the up-regulated genes numbered 1362, 2227, and 172, while the down-regulated genes numbered 2373, 3708, and 340, respectively. Differential gene expression plots (Figure 5A) revealed the presence of unique genes for each of the three comparative groups: O vs. G, R vs. G, and R vs. O, with counts of 747, 2759, and 149, respectively.

The identified differentially expressed genes underwent Gene Ontology (GO) functional enrichment analysis utilizing the ClusterProfiler software (version 4.14.4). The analysis categorized these genes into the components of biological process (BP), cellular component (CC), and molecular function (MF), employing an adjusted *p*-value (padj) threshold of less than 0.05 to determine significant enrichment. The total numbers of differentially expressed genes enriched by GO analysis in the comparisons of O vs. G, R vs. G, and R vs. O were 3632, 5613, and 465, respectively. The enriched differential genes comprised 500, 588, and 289 genes identified through Biological Process (BP); 87, 123, and 16 genes identified through Cellular Component (CC); and 336, 397, and 176 genes identified through Molecular Function (MF). In the comparative analyses of O vs. G and R vs. G, within the Biological Process (BP) category, the three Gene Ontology (GO) terms exhibiting the highest enrichment of differential genes were movement of cell or subcellular component (GO:0006928), microtubule-based movement (GO:0007018), and microtubule-based process (GO:0007017). In the Cellular Component (CC) category, the three GO terms with the greatest abundance in differential gene enrichment were extracellular region (GO:0005576), cell wall (GO:0005618), and external encapsulating structure (GO:0030312). The three most GO terms associated with differential gene enrichment in the Molecular Function (MF) category are microtubule binding (GO:0008017), microtubule motor activity (GO:0003777), and tubulin binding (GO:0015631). In the comparison between R and O, the GO terms most significantly enriched for differential genes within the Biological Process (BP) category are the isoprenoid metabolic process (GO:0006720), isoprenoid biosynthetic process (GO:0008299), and cellular lipid metabolic process (GO:0044255); GO terms associated with the Cellular Component (CC) category are as follows: preribosome (GO:0030684), nucleolus (GO:0005730), and the eukaryotic translation initiation factor 3 complex (GO:0005852). Within the Molecular Function (MF) category, the GO terms include oxidoreductase activity, acting on paired donors with incorporation or reduction in molecular oxygen (GO:0016705), O-methyltransferase activity (GO:0008171), and iron ion binding (GO:0005506). By comparing the KEGG database, differentially expressed genes were enriched into various metabolic pathways. As shown in Figure 5B–D, flavonoid biosynthesis (ko00941) was enriched in all three comparison combinations.

### 2.4. Combined Analysis of Integrated Transcriptomics and Metabolomics

**Figure 6:**
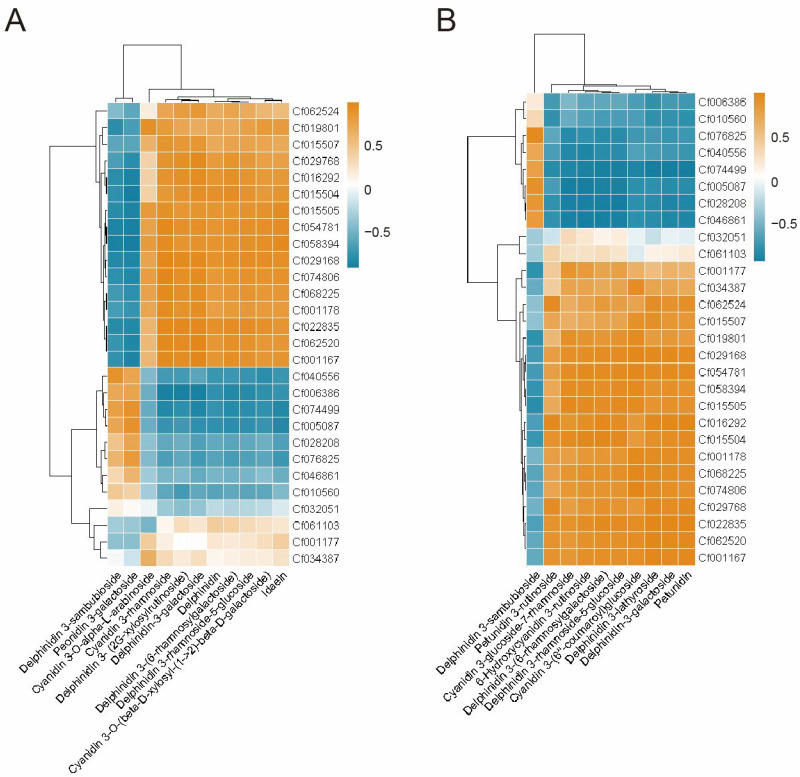
Heatmap of the correlation between differential genes and differential anthocyanin metabolites. (**A**) O versus G. (**B**) R versus G.

The correlation between genes and metabolites was plotted in a clustered heat map (Table 1). As shown in Figure 6, the comparative analysis of O vs. G revealed that Delphinidin 3-sambubioside exhibited a positive correlation with a total of ten genes; specifically, delphinidin 3-sambubioside was associated with *CfCHS3_1*, *CfCHS3_2*, *CfCHS3_3*, *CfF3H_3*, *CfCYP75B2_1*, *CfCYP75A6_1*, *CfCYP75A6_2*, *CfDFRA*, *CfANS*, and *Cf3GGT*. A more significant positive correlation was observed with five specific genes: *CfCHS3_3*, *CfF3H_3*, *CfCYP75A6_1*, *CfDFRA*, and *Cf3GGT*. Peonidin 3-galactoside exhibited a positive correlation with the genes *CfCHS3_1*, *CfCHS3_2*, *CfCHS3_3*, *CfF3H_3*, *CfCYP75A6_1*, *CfCYP75A6_2*, *CfDFRA*, *CfANS*, and *Cf3GGT*. Among these, a more substantial positive correlation was noted with six genes: *CfCHS3_1*, *CfCHS3_3*, *CfF3H_3*, *CfCYP75A6_1*, *CfDFRA*, and *Cf3GGT*. Both differential metabolites were significantly negatively correlated with the *4CL* and *CHI* gene categories. In the R vs. G comparison, delphinidin 3-sambubioside showed a positive correlation with eight genes: *CfCHS3_1*, *CfCHS3_2*, *CfCHS3_3*, *CfF3H_3*, *CfCYP75A6_1*, *CfDFRA*, *CfANS*, and *Cf3GGT*. It also positively correlated with six genes: *CfCHS3_1*, *CfCHS3_2*, *CfCHS3_3*, *CfF3H_3*, *CfDFRA*, and *Cf3GGT*, while maintaining a significant negative correlation with *4CL* and *CHI* genes. The biological pathway of anthocyanin synthesis was further elucidated by integrating the metabolic pathway map with a heat map (Figure 6). Analysis of these results indicated that *CfCHS3_1*, *CfCHS3_2*, *CfCHS3_3*, *CfF3H_3*, *CfCYP75A6_1*, *CfDFRA*, and *Cf3GGT* predominantly contribute to the anthocyanins delphinidin 3-sambubioside and peonidin 3-galactoside.

### 2.5. qRT-PCR Validation Results

**Figure 7:**
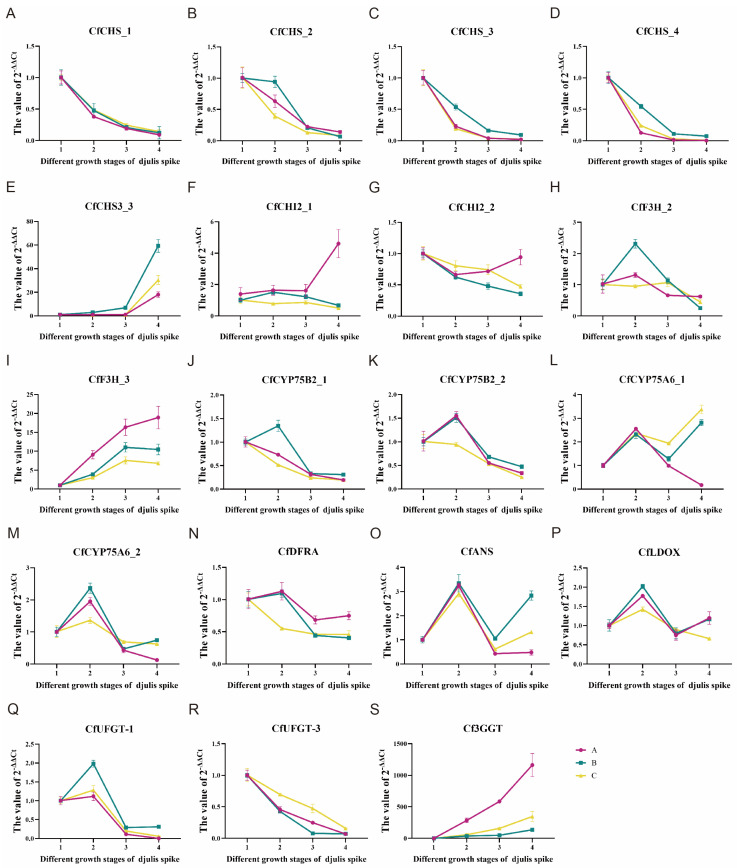
Line graph of real-time PCR analysis of anthocyanin biosynthesis-related genes in the spike of djulis. 1–4 represent the 20th, 30th, 35th, and 40th days after the end of the full flowering period, respectively. A B, and C represent three biological replicates. (**A**) *CfCHS_1*(*Cf062520*, chalcone synthase). (**B**) *CfCHS_2*(*Cf001167*, chalcone synthase). (**C**) *CfCHS_3*(*Cf001178*, chalcone synthase). (**D**) *CfCHS_4*(*Cf062524*, chalcone synthase). (**E**) *CfCHS3_3*(*Cf076825*, chalcone synthase 3). (**F**) *CfCHI2_1*(*Cf054781*, chalcone isomerase). (**G**) *CfCHI2_2*(*Cf022835*, chalcone isomerase). (**H**) *CfF3H_2*(*Cf016292*, flavanone 3-hydroxylase). (**I**) *CfF3H_3*(*Cf040556*, flavanone 3-hydroxylase). (**J**) *CfCYP75B2_1*(*Cf034387*, flavonoid 3′-monooxygenase). (**K**) *CfCYP75B2_2*(*Cf058394*, flavonoid 3′-monooxygenase). (**L**) *CfCYP75A6_1*(*Cf006386*, flavonoid 3′,5′-hydroxylase). (**M**) *CfCYP75A6_2*(*Cf032051*, flavonoid 3′,5′-hydroxylase). (**N**) *CfDFRA*(*Cf074499*, dihydroflavonol 4-reductase). (**O**) *CfANS*(*Cf010560*, anthocyanidin synthase). (**P**) *CfLDOX*(*Cf061103*, leucoanthocyanidin dioxygenase). (**Q**) *CfUFGT_1*(*Cf015504*, anthocyanidin 3-O-glucosyltransferase). (**R**) *CfUFGT_3*(*Cf015507*, anthocyanidin 3-O-glucosyltransferase). (**S**) *Cf3GGT* (*Cf005087*, anthocyanidin 3-O-glucoside 2″-O-glucosyltransferase).

To evaluate the gene expression analysis, 20 enriched genes were selected for validation using qRT-PCR. As shown in Figure 7, these genes include six *CHS*s (*Cf046861*, *Cf076825*, *Cf062520*, *Cf001167*, *Cf001178*, *Cf062524*), two *CHI*s (*Cf054781*, *Cf022835*), two *F3H*s (*Cf016292*, *Cf040556*), two *CYP75B2*s (*Cf034387*, *Cf058394*), two *CfCYP75A6*s (*Cf006386*, *Cf032051*), one *DFRA* (*Cf074499*), one *ANS* (*Cf010560*), one *LDOX* (*Cf061103)*, two *UFGT*s (*Cf015504*, *Cf015507*), and one *3GGT* (*Cf005087*). The qRT-PCR analysis demonstrated that the expression levels of *CHS*, *CHI*, *F3H*, *CYP75B2*, and *UFGT* genes agreed with the transcriptomic data across the four developmental stages from green to red. Notably, the genes *CfCHS3_2*, *CfCHS3_3*, *CfF3H_3*, *CfCYP75A6_1*, and *Cf3GGT* exhibited a continuous up-regulation trend. Conversely, the expression of *CfCHS_1*, *CfCHS_2*, *CfCHS_3*, *CfCHS_4*, *CfCHI2_1*, *CfCHI2_2*, *CfF3H_2*, *CfCYP75B2_1*, *CfCYP75B2_2*, and *CfUFGT_3* was progressively downregulated, corroborating the transcriptomic analysis findings. *CfDFRA* exhibited a pattern of rising and then falling, ultimately showing a downward trend that did not align with the upregulation observed in the transcriptomics data. *CfANS* displayed a pattern of rising, then falling, and rising again, with an overall upward trend in expression, aligning with the transcriptomics analysis. *CfCYP75A6_2* and *CfLDOX* showed a pattern of rising, then falling, and rising again, with an overall downward trend, which was generally consistent with the transcriptomics results. The expression pattern of *CfUFGT_1* exhibited an initial increase followed by a decrease, ultimately demonstrating an overall declining trend. This pattern was largely consistent with the findings from the transcriptomics analysis.

In conclusion, the qRT-PCR results for 15 out of the 20 selected genes aligned with the transcriptomics analysis, successfully fulfilling the experimental objectives. In the qRT-PCR experiments, a gradual transition from green to red was incorporated, along with a refinement of the red phase at various maturation stages, revealing a dynamic gene expression process. The findings were largely consistent with the transcriptome results, except for *CfDFRA*, which deviated from the transcriptomic data.

## 3. Discussion

### 3.1. The Biosynthesis of Anthocyanins in the Spike of Djulis

Natural pigments derived from natural sources are integral to the vibrant coloration observed in various plant species. Among these pigments, anthocyanins, betalains, and carotenoids have garnered significant research interest due to their distinctive biological properties and their role in plant coloration mechanisms. Nonetheless, there remains an absence of pertinent studies concerning the key structural genes implicated in anthocyanin biosynthesis within the spikes of djulis. To elucidate the presence of anthocyanins and the potential molecular mechanisms underlying their formation in the spicules of djulis, this study employed an integrated approach combining metabolomic and transcriptomic analyses. This comprehensive examination was conducted on spicule samples of djulis collected across various color developmental stages.

Through the detection of anthocyanin content in the spike of djulis, targeted anthocyanin metabolomics analysis, and non-targeted metabolomics analysis, we found that there are significant changes in anthocyanin during the process of transitioning from green to red or orange. Specifically, during the process of djulis from the flowering stage to the maturation stage, most of the anthocyanin content increases, and only a small portion of anthocyanin content accumulates. We have identified two types of anthocyanins, delphinidin 3-sambubioside and peonidin 3-galactoside, that accumulate during the process of transitioning from green to red or orange. A combined analysis of transcriptomics and targeted metabolomics further elucidated differential anthocyanins and key genes that facilitate anthocyanin biosynthesis, predominantly active during the color transition phase. Through transcriptomic analysis, we identified 28 differentially expressed genes associated with anthocyanin biosynthesis. These include 2 *4-coumarate-CoA ligases* (*4CL*s), 9 *chalcone synthases* (*CHS*s), 3 *chalcone isomerases* (*CHI*s), 3 *flavanone 3-hydroxylases* (*F3H*s), 2 *flavonoid 3′-hydroxylases* (*F3′H*s), 2 *flavonoid 3′,5′-hydroxylases* (*F3′5’H*s), 1 *dihydroflavonol 4-reductase* (*DFR*), 1 *anthocyanidin synthase* (*ANS*), 1 *leucoanthocyanidin dioxygenase* (*LDOX*), 3 *UDP-glucose: flavonoid 3-O-glucosyltransferases* (*UFGT*s), and 1 *anthocyanin 3-O-glucoside-6″O-malonyltransferase* (*3GGT*). Concurrently, targeted metabolomic analysis revealed 17 differentially expressed anthocyanin metabolites. Among these, delphinidin 3-sambubioside and peonidin 3-galactoside were identified as the primary metabolites exhibiting up-regulated expression. The correlation analysis between differential genes and metabolites revealed that seven genes, specifically three *CHS*s (*CfCHS3_1*, *CfCHS3_2*, *CfCHS3_3*), one *F3H* (*CfF3H_3*), one *F3’5’H* (*CfCYP75A6_1*), one *DFR* (*CfDFRA*), and one *3GGT* (*Cf3GGT*), exhibited significant positive correlation with the up-regulated anthocyanins, delphinidin 3-sambubioside and peonidin 3-galactoside. Conversely, the genes *4CL* and *CHI* showed significant negative correlations with these anthocyanins. Consequently, it is highly probable that the seven genes serve as the principal structural genes governing the biosynthesis of delphinidin 3-sambubioside and peonidin 3-galactoside.

*CHS* serves as a pivotal enzyme in the initial crucial step of the flavonoid biosynthesis pathway [53]. Similarly, *F3H* represents a significant enzyme class involved in the formation of dihydroflavonols and is regarded as a key enzyme at the branching point of the flavonoid biosynthesis pathway [54]. Furthermore, *F3′5′H* facilitates the production of delphinidin [26], corroborating the findings of our study, which demonstrated that the synthetic pathway involving *F3′5′H* resulted in delphinidin production. *DFR* plays a crucial role in the catalysis of anthocyanin biosynthesis by reducing dihydroflavonols to colorless anthocyanidins [55]. According to Cheng et al. [56], *3GGT* is responsible for the subsequent glycosylation of anthocyanin 3-O-glucosides, a process that not only stabilizes anthocyanins but also facilitates their transport into vesicles. Ueyama et al. [57] reported that delphinidin is the predominant anthocyanidin in *Nierembergia* sp. and that the genes *CHS*, *F3′5’H*, and *DFR* are coordinately regulated in relation to delphinidin accumulation. Similarly, Suzuki et al. [58] observed in their investigation of perianth pigmentation in Asiatic hybrid lilies (*Lilium* spp.) that the expression levels of *CHS* and *DFR* were significantly elevated in pigmented regions containing anthocyanins compared to unpigmented parts. In their study on the purple pods of *Lablab purpureus* (L.) Sweet, Cui et al. [59] identified five anthocyanin derivatives, specifically those of malvidin, delphinidin, and petunidin, with delphinidin derivatives being the most prevalent. Furthermore, the expression levels of the genes *PAL*, *F3H*, *F3’H*, *DFR*, and *ANS* were significantly increased in the purple pods compared to the green pods. In the research conducted by Shi et al. [60], the results showed that *CHS* and *DFR* are crucial in influencing the pigmentation of *Paeonia rockii*, primarily by modulating the ratio of cyanidin to peonidin, thereby resulting in dark purple or purple-red hues. Similarly, a study by Mei et al. [61] demonstrated that *3GT* expression was significantly up-regulated, contributing to the accumulation of delphinidin and cornflowerin in the “Zijuan” tea plant (*Camellia sinensis* var. assamica). The study by Zhang et al. [62] highlighted the role of *CHS* genes in the development of pink pigmentation in quinoa leaves. Our findings corroborate these results, demonstrating that *CHS*, *F3H*, *F3′5′H*, *DFR*, and *3GGT* are integral to the biosynthesis of anthocyanins.

**Figure 8:**
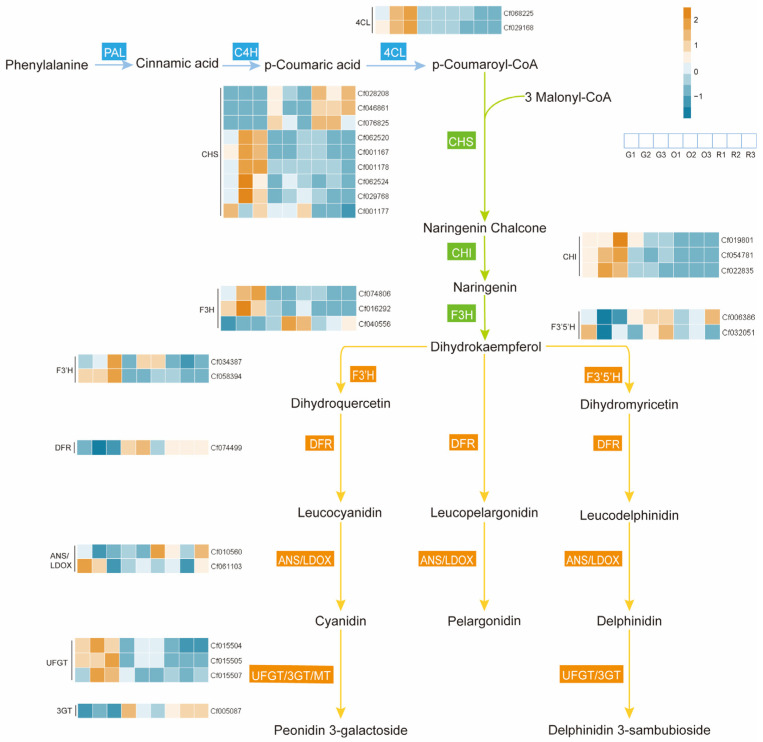
Anthocyanin biosynthesis pathway in the spike of djulis. PAL: phenylalanine ammonialyase; C4H: cinnamate-4-hydroxylase; 4CL: 4-Coumarate-CoA ligase; CHS: chalcone synthase; CHI: chalcone isomerase; F3H: flavanone 3-hydroxylase; F3′H: flavonoid 3′-hydroxylase; F3’5’H: flavonoid 3’,5’-hydroxylase; DFR: dihydroflavonol 4-reductase; ANS: anthocyanidin synthase; LDOX: leucoanthocyanidin dioxygenase; UFGT: UDP-glucose flavonoid 3-O-glucosyl transferase; GT: glucosyltransferase.

Based on the results of this experiment, we propose the potential presence of anthocyanins in the spikes of djulis (Figure 8). We hypothesize that the high expression of the genes *CfCHS3_1*, *CfCHS3_2, CfCHS3_3*, *CfF3H_3*, *CfCYP75A6_1*, *CfDFRA*, and *Cf3GGT* are pivotal in the biosynthesis of the two anthocyanins, delphinidin 3-sambubioside and peonidin 3-galactoside. Our findings offer a molecular theoretical foundation for understanding the mechanism underlying spike color formation in djulis. This study exclusively investigated the structural genes associated with the biosynthesis of delphinidin 3-sambubioside and peonidin 3-galactoside. Currently, it remains undetermined whether these anthocyanins contribute to the coloration of djulis spikes, warranting further investigation in this context.

### 3.2. The Change in Spike Color May Be the Result of the Combined Action of Anthocyanins and Betalains

Although the content of betalains in the spike of djulis was not determined in this study, a noteworthy finding in our study is that the betalains and anthocyanin were identified in the metabolites through non-targeted metabolomics. Djulis and quinoa, which are closely related crops in the genus *Quinoa* of the *Amaranthaceae* family, exhibit similar biological properties [63,64]. Our non-targeted metabolomic and targeted anthocyanin metabolomic analyses revealed the presence of anthocyanins in the spikes of djulis. Gorinstein et al. [65] and Paśko et al. [66] conducted studies to characterize the antioxidant activity of quinoa seeds and shoots by quantifying compounds such as total phenolics and anthocyanins. In a subsequent study, Zhang et al. [62] elucidated the biosynthetic pathway of anthocyanins in quinoa leaves, indicating that anthocyanins are present in the leaves and contribute to their pink coloration. The results of this experiment and previous research suggest the presence of anthocyanins in *Quinoa* spp. However, in previous studies, it has also been reported that betalains are the compounds responsible for the pigmentation in quinoa and djulis [23,67,68]. Many studies have mentioned the mutual exclusivity of anthocyanins and betalains [35,69]. In their investigation of betalain biosynthesis and its evolution in purple jasmine (*Mirabilis jalapa* L.), Polturak et al. [70] discovered that genes associated with anthocyanin synthesis, especially anthocyanin synthase (*MjANS*), exhibit high expression levels in petals accumulating betalains. However, the synthesis of anthocyanin is impeded due to the absence of the requisite active site region. Furthermore, a study conducted by Pucker et al. [71] proposed several mechanisms to elucidate the reduction in anthocyanins in beet-pigmented Caryophyllales. These mechanisms primarily involve the loss of a key enzyme, the downregulation of synthesis genes, and the degeneration of regulatory complexes. Consequently, the current experiment detected the presence of both anthocyanins and betalains, suggesting a potential coexistence of these pigments. Lu et al. [72], in their investigation into the impact of nitrogen non-thermal pressure plasma on the natural bioactive compounds in djulis seeds, observed a significant increase in the levels of betacyanin and anthocyanin in seeds treated with nitrogen non-thermal plasma. These findings align with the concurrent detection of anthocyanins and betacyanins reported in the present study. The longstanding notion of the non-coexistence and mutual exclusivity of anthocyanins and betacyanins has been well-documented. This study provides a significant case for further investigation into the potential coexistence of these pigments.

## 4. Materials and Methods

### 4.1. Plant Materials

**Figure 9:**
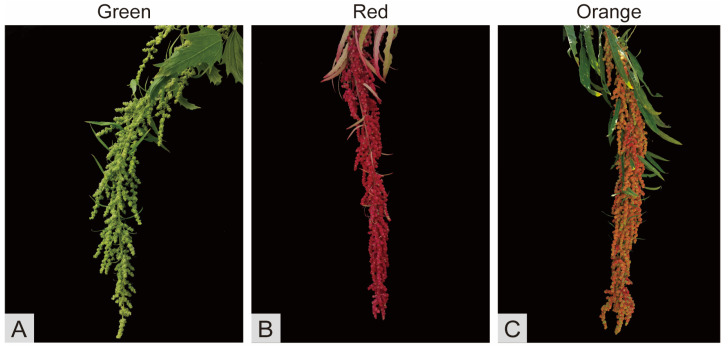
Djulis in different color periods. (**A**) The spike of djulis during the green period before maturity. (**B**) The spike of djulis during the red period after maturity. (**C**) The spike of djulis during the orange period after maturity.

The experimental material used in this study was djulis, sourced from Chengdu University. Seven different developmental stages, ranging from the onset of full bloom to complete maturity, were selected to determine the anthocyanin content. Samples for omics analysis were specifically selected from green spikes at the mid-developmental stage, as well as from red and orange spikes at the mature stage (Figure 9). For the convenience of description in the article, these three kinds of samples were labeled as green (G), red (R), and orange (O), respectively.

### 4.2. Determination of Anthocyanin Content

To ascertain the presence of anthocyanins in the spikes, we collected samples from seven distinct developmental stages, specifically from the full bloom stage to 15, 20, 25, 30, 35, and 40 days after pollination (DAP). Anthocyanin content from each sample was detected utilizing the Leagene anthocyanin test kit (Leagene. Beijing, China) [62,73]. Firstly, 0.25 g of the sample from each developmental stage was accurately weighed and mixed with 8 mL of a 0.1 mol/L hydrochloric acid methanol solution. The samples were then enveloped in aluminum foil and stored at 4 °C, shielded from light, overnight to allow the sample tissue to turn white. Following this, the supernatant was obtained by centrifugation at 9569× *g* (cence, Changsha, China) for ten minutes. A volume of 200 µL of the extraction solution was aspirated using a sterile pipette and transferred into the wells of a 96-well plate. The acidic methanol extraction solution served as a blank control. The absorbance at 530 nm was subsequently measured using a BioTek SYNERGY HTX microplate reader (BioTek, Winooski, America). Definition of Anthocyanin Unit: An anthocyanin unit (U) is defined as the concentration of anthocyanin corresponding to an optical density (OD) value of 0.1. Calculation Method: The anthocyanin content in tissue samples, expressed as units per gram (U/g), is calculated using the formula: (A_sample − A_blank)/(0.1 × 0.25 g), where A_sample represents the OD value of the anthocyanin extract measured at 530 nm.

### 4.3. Anthocyanins Targeted Metabolomics Analysis

The materials used for targeted metabolomics detection were green (G), red (R), and orange (O), with three biological replicates for each color of spike. The samples were accurately weighed, and 1000 μL of 60% methanol (containing 0.1 mol/L hydrochloric acid and 0.1% ethylenediaminetetraacetic acid disodium salt by mass) was added and vortexed for 60 s. The samples were added with steel beads and then put into a tissue grinder and ground at 50 Hz for 120s. The samples were ultrasonicated at 40 °C for 10 min with 60% ultrasound power and then centrifuged at 4 °C for 10 min at 13,780× *g*. The supernatant was filtered through a 0.22 μm membrane, and the filtrate was added to the assay bottle for LC-MS/MS detection.

The raw mass spectra were converted to mzML file format using the MS Convert tool in the Proteowizard package (v3.0.8789). Peak detection, peak filtering, and peak alignment were performed using the XCMS software (version 1.42.0) package to obtain a list of peak areas of substances. The data were batch normalized for peak area to allow comparison of different magnitudes. The public databases KNApSAcK (http://www.knapsackfamily.com/KNApSAcK/ (accessed on 9 January 2025)), HMDB (http://www.hmdb.ca/ (accessed on 9 January 2025)), LipidMaps, PubChem, and KEGG were used for the identification of the substances, and the parameters were set to ppm < 30 ppm. The total peak normalization method was used to achieve data correction and eliminate systematic errors. The Ropls from the R software package (version 3.3.2) were used to perform principal component analysis (PCA), partial least squares-discriminant analysis (PLS-DA), and orthogonal partial least squares-discriminant analysis (OPLS-DA) for dimensionality reduction in the sample data. Score plots, loading plots, and S-plot plots were plotted to demonstrate the differences in anthocyanin composition among the samples. Based on the statistical test to calculate the *p*-value, the OPLS-DA dimensionality reduction method to calculate the variable projection importance (VIP), and Fold Change to calculate the multiplicity of differences between groups, we measured the intensity of the influence of the content of each anthocyanin component on the classification discrimination of the samples and the explanatory ability, which assisted in the screening of marker anthocyanins. The screening threshold was *p*-value ≤ 0.05 and VIP ≥ 1.

### 4.4. Non-Targeted Metabolomics Analysis

The materials used for the non-targeted metabolome assay comprised 18 djulis spike tissue samples, with an equal distribution of six samples each for the green (G), red (R), and orange (O) categories. The samples were accurately weighed to 200 mg (±1%) and prepared by the addition of 0.6 mL of 2-chlorophenylalanine (4 ppm) dissolved in methanol at −20 °C. The mixture was subjected to vortexing and oscillation for 30 s. Subsequently, 100 mg of glass beads were incorporated, and the samples were processed using a TissueLysis II tissue grinder (Thermo, Waltham, America) at a frequency of 25 Hz for 60 s. The sample was subjected to ultrasonication at room temperature for 15 min. Subsequently, centrifugation was performed at 1750× *g* for 10 min at 25 °C. A volume of 300 μL of the resulting supernatant was then filtered through a 0.22 μm membrane. The filtrate was transferred into assay vials, and 20 μL from each sample was combined to create quality control (QC) samples. The remaining samples were used for the liquid chromatography-mass spectrometry (LC-MS) analysis.

The raw data collected were converted into the mzXML format (xcms input file format) by ProteoWizard software (version 3.0.8789). Subsequent processes of peak identification, filtering, and alignment were performed using the XCMS package for R (version 3.3.2). This resulted in the generation of a data matrix containing detailed information on mass-to-charge ratios, retention times, and peak areas. To facilitate the comparison of datasets with varying magnitudes, batch normalization of the peak area data was performed. Multivariate statistical analyses, including principal component analysis (PCA), partial least squares discriminant analysis (PLS-DA), and orthogonal-partial least squares discriminant analysis (OPLS-DA), were conducted using the SIMCA-P software package (version 13.0) and the ropls package within the R programming language. The *p*-value was calculated according to the statistical test, while the variable importance in projection (VIP) was calculated through the OPLS-DA dimensionality reduction method. Fold Change (FC) was used to evaluate the magnitude of differences between groups, indicating the influence and explanatory power of each metabolite’s content on sample classification. These metrics aided in identifying marker metabolites, with differential metabolites screened using thresholds of *p*-value ≤ 0.05 and VIP ≥ 1.

### 4.5. Transcriptomics Analysis

The samples used for transcriptome analysis were green (G), red (R), and orange (O). RNA was extracted from the samples using kits (Tengen polysaccharide polyphenol kit, QIAGEN, Dusseldorf, Germany). Rigorous quality control of RNA samples was performed with the Agilent 2100 bioanalyzer to accurately detect RNA integrity. Subsequently, cDNA libraries were constructed for Illumina sequencing, with three biological replicates performed for each group.

Following quality control, the clean reads were efficiently and accurately aligned to the hexaploid djulis reference genome (accessible at https://www.ncbi.nlm.nih.gov/bioproject/840947 (accessed on 9 January 2025)) using the HISAT2 software (version 2.0.5). Subsequently, novel transcripts were assembled using StringTie software (version 1.3.3b). These newly assembled transcripts were then annotated by retrieving information from various databases, including Pfam (http://pfam.xfam.org/ (accessed on 9 January 2025)), SUPERFAMILY (https://supfam.org/SUPERFAMILY/ (accessed on 9 January 2025)), GO (https://www.geneontology.org/ (accessed on 9 January 2025)), and KEGG (https://www.genome.jp/kegg/ (accessed on 9 January 2025)) to obtain annotation information.

### 4.6. Differential Genes Identification, GO, and KEGG Enrichment Analysis

Raw read count values were obtained by quantitative analysis using the FeatureCounts tools in the Subread software suite (version 1.5.0-p3). Gene expression levels were quantified using Fragments Per Kilobase Million (FPKM) to account for variations in sequencing depth and gene length. Differential gene expression analysis was conducted using the DESeq2 software (version 1.20.0), wherein the raw read counts were initially normalized, followed by the application of a statistical model to compute the probability values (*p*-values) for hypothesis testing. Finally, corrections for multiple hypothesis testing were conducted to calculate the false discovery rate (FDR), with the adjusted *p*-value (padj) being a commonly used metric. The criteria for identifying differentially expressed genes were set as |log2 (FoldChange)| ≥ 1 and padj ≤ 0.05.

After obtaining differential genes based on gene expression scores, gene functions will be enriched for analysis. The enrichment analysis is based on the principle of hypergeometric distribution, where the set of differential genes is the set of differential genes obtained from differential significant analysis and annotated to the GO or KEGG database. The software used was Cluster Profiler software (version 3.8.1). The GO function enrichment and KEGG enrichment analyses were performed using the GO database (https://www.geneontology.org/ (accessed on 9 January 2025)) and the KEGG database (https://www.genome.jp/kegg/ (accessed on 9 January 2025)), respectively. GO function enrichment and KEGG pathway enrichment were performed with a padj ≤ 0.05 as the threshold for being a significant enrichment.

### 4.7. Combined Transcriptomics and Metabolomics Analysis

Transcriptomic and metabolomic data were integrated to investigate the interactions between genes and metabolites. The Pearson correlation coefficient (PCC) method was employed to calculate the correlation coefficients between the selected genes and metabolites. A PCC value of 0.6 or higher was considered indicative of a significant correlation between the genes and metabolites.

### 4.8. qRT-PCR Validation

Four stages of djulis spike tissues were selected at the 20th, 30th, 35th, and 40th days after the end of the full flowering period. These stages were designated as periods 1, 2, 3, and 4, respectively. For each period, three biological replicates were prepared. RNA was extracted from each sample and subsequently reverse-transcribed to synthesize complementary DNA (cDNA) using the Mei5bio kit. This cDNA served as a template for quantitative reverse transcription polymerase chain reaction (qRT-PCR) verification. The experimental protocol involved an initial pre-denaturation step at 95 °C for 1 min, followed by denaturation at 95 °C for 15 s, annealing at 60 °C for 15 s, and extension at 72 °C for 30 s, across a total of 40 cycles. *UBI3* was selected as the internal reference gene. The coding sequence (CDS) of the target gene, as identified in the annotation file, was utilized in conjunction with SnapGene version 6.0.2 and the NCBI Primer Design tool (https://www.ncbi.nlm.nih.gov/tools/primer-blast (accessed on 9 January 2025)). The relative expression levels of the target genes were quantified using the 2^−ΔΔCt^ method [74].

### 4.9. Statistical Analysis

Data analysis and plotting for each histology section were analyzed and generated using R packages, including ropls, reshape2, ggplot2, and heatmap in Rstudio software (version 4.2.2). Additionally, qRT-PCR results were analyzed and visualized using GraphPad Prism version 10.2.2 software. The results were calculated in x ± n. The RNA-seq data were submitted to the NCBI BioProjects database (accession number: PRJNA1199591).

## 5. Conclusions

The integrated transcriptomic and metabolomic analysis revealed that there are alterations in anthocyanin level changes during the transition of spike color from green to red or orange in djulis, both prior to and following maturation. The metabolites associated with anthocyanin synthesis were predominantly enriched in the flavonoid biosynthesis pathway (ko00941) and the anthocyanin biosynthesis pathway (ko00942). Two anthocyanins, delphinidin 3-sambubioside and peonidin 3-galactoside, progressively accumulated during the transition of spike color from green to red or orange in djulis, and the high expression levels of *CfCHS3_1*, *CfCHS3_2*, *CfCHS3_3*, *CfF3H_3*, *CfCYP75A6_1*, *CfDFRA*, and *Cf3GGT* are pivotal in facilitating the synthesis of these two anthocyanins. Our findings provide a foundation for exploring the potential coexistence of anthocyanins and betalains.

## Figures and Tables

**Table 1:** Anthocyanin and flavonoid biosynthesis of putative unigenes involved in spike color variation.

Unigene_Id	Short Gene Name	Full Gene Name
Cf068225	Cf4CL2_1	4-Coumarate-CoA ligase 2
Cf029168	Cf4CL2_2	4-Coumarate-CoA ligase 2
Cf062520	CfCHS_1	Chalcone synthase
Cf001167	CfCHS_2	Chalcone synthase
Cf001178	CfCHS_3	Chalcone synthase
Cf062524	CfCHS_4	Chalcone synthase
Cf029768	CfCHS_5	Chalcone synthase
Cf028208	CfCHS3_1	Chalcone synthase 3
Cf046861	CfCHS3_2	Chalcone synthase 3
Cf076825	CfCHS3_3	Chalcone synthase 3
Cf001177	CfCHS3_4	Chalcone synthase 3
Cf019801	CfCHI_1	Chalcone-flavanone isomerase
Cf054781	CfCHI2_1	Chalcone isomerase
Cf022835	CfCHI2_2	Chalcone isomerase
Cf074806	CfF3H_1	Flavanone 3-hydroxylase
Cf016292	CfF3H_2	Flavanone 3-hydroxylase
Cf040556	CfF3H_3	Flavanone 3-hydroxylase
Cf034387	CfCYP75B2_1	Flavonoid 3′-monooxygenase
Cf058394	CfCYP75B2_2	Flavonoid 3′-monooxygenase
Cf006386	CfCYP75A6_1	Flavonoid 3′,5′-hydroxylase
Cf032051	CfCYP75A6_2	Flavonoid 3′,5′-hydroxylase
Cf074499	CfDFRA	Dihydroflavonol 4-reductase
Cf010560	CfANS	Anthocyanidin synthase
Cf061103	CfLDOX	Leucoanthocyanidin dioxygenase
Cf015504	CfUFGT_1	Anthocyanidin 3-O-glucosyltransferase
Cf015505	CfUFGT_2	Anthocyanidin 3-O-glucosyltransferase
Cf015507	CfUFGT_3	Anthocyanidin 3-O-glucosyltransferase
Cf005087	Cf3GGT	Anthocyanidin 3-O-glucoside 2″-O-glucosyltransferase

## Data Availability

The raw RNA-seq data were submitted to the NCBI BioProjects database (accession number: PRJNA1199591).
